# Supplementary figures and images for: Restoring South African subtropical succulent thicket using Portulacaria afra: rooting variation across three soil types
Source: PeerJ. 2025 Jun 9;13:e19303. doi: 10.7717/peerj.19303 (PMC12161136; doi:10.7717/peerj.19303)

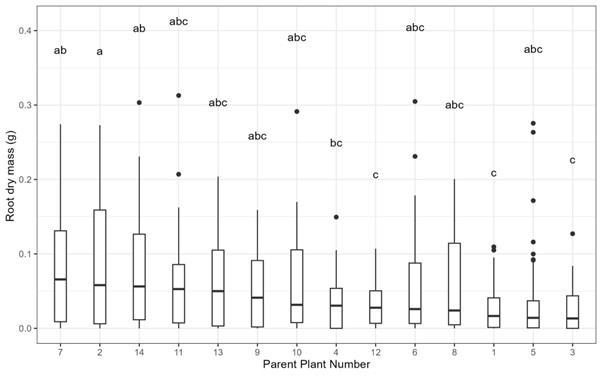

Supplement: Supplemental Information 4 — There was a significant difference in rooting across the parent plants (ANOVA: F13,564 = 4.141, p < 0.001) and dissimilar superscripts indicate significant differences between plants (post-hoc Tukey test). [file peerj-13-19303-s004.jpg]
