# Supplementary material for: Restoring South African subtropical succulent thicket using Portulacaria afra: rooting variation across three soil types
Source: PeerJ. 2025 Jun 9;13:e19303. doi: 10.7717/peerj.19303 (PMC12161136; doi:10.7717/peerj.19303)
Supplement: Supplemental Information 9 [file peerj-13-19303-s009.docx]

| **Site** | **A** | **B** | **C** |
| --- | --- | --- | --- |
| Latitude | S33.52111 | S33.47586 | S33.47609 |
| Longitude | E25.38139 | E25.34640 | E 25.34516 |
| Lab No. | SL22-20941 | SL22-20943 | SL22-20942 |
| pH (KCl) | 6.5 | 6.8 | 7.9 |
| Resist. (ohm) (saturated paste) | 270 | 420 | 1030 |
| Electrical conductivity (mS.m^-1^) (saturated paste) | 370 | 238 | 97 |
| Electrical conductivity (mS.m^-1^) (1:5)† | 46-51 | 30-33 | 12-13 |
| Stone (Vol %) | 1.03 | 1.93 | 2.98 |
| NO_3_ (mg/kg) | 98.9 | 22.1 | 4.7 |
| P (Bray I) (mg/kg) | 94.2 | 36.7 | <0.77 |
| P (Olsen) (mg/kg) |  |  | 5.9 |
| P(Bray ll) (mg/kg) | 116 | 43.4 | <2.2 |
| NH_4_ (mg/kg) | 4.2 | 1.6 | 2.1 |
| K (mg/kg) | 814 | 1050 | 342 |
| Ca^2+^ (cmol(+)/kg) | 8.8 | 6.4 | 25.4 |
| Mg^2+^ (cmol(+)/kg) | 2.4 | 2.5 | 4.0 |
| K^+^ (cmol(+)/kg) | 2.1 | 2.7 | 0.88 |
| Na^+^ (cmol(+)/kg) | 0.98 | 0.44 | 0.32 |
| Cu (mg/kg) | 2.1 | 1.5 | <0.33 |
| Zn (mg/kg) | 3.0 | 1.6 | <0.36 |
| Mn (mg/kg) | 193 | 165 | 2.0 |
| B (mg/kg) | 0.69 | 0.83 | 1.5 |
| Fe (mg/kg) | 268 | 163 | <0.33 |
| C (%) | 1.52 | 0.68 | 1.31 |
| S Am.acet (mg/kg) | 18.2 | 6.7 | 16.4 |
| C (Leco) (%) | 1.88 | 1.08 | 5.83 |
| CEC (cmol/kg) | 11.42 | 9.26 | 6.74 |
| Na saturation (%) | 6.86 | 3.65 | 1.05 |
| K saturation (%) | 14.62 | 22.36 | 2.87 |
| Ca saturation (%) | 61.62 | 53.16 | 83.01 |
| Mg saturation (%) | 16.81 | 20.76 | 13.07 |
| T Value (cmol/kg) | 14.28 | 12.04 | 30.60 |
| Acid Sat. (%) | 0.00 | 0.00 | 0.00 |
| Clay (%) | 20.0 | 16.0 | 10.0 |
| Silt (%) | 24.0 | 22.0 | 18.0 |
| Sand (%) | 56.0 | 62.0 | 72.0 |
| Fine Sand (%) | 44.1 | 49.8 | 49.2 |
| Medium Sand (%) | 9.7 | 5.3 | 11.3 |
| Coarse Sand (%) | 2.2 | 6.9 | 11.5 |
| Stone (% (v/v)) | 0.6 | 1.1 | 1.7 |
| Classification | SANDY CLAY LOAM | FINE SANDY LOAM | FINE SANDY LOAM |
| Waterholding (10kPa %) | 34.93 | 34.75 | 30.28 |
| Waterholding (100kPa %) | 21.49 | 20.42 | 16.91 |
| Waterholding (mm/m) | 134.42 | 143.35 | 133.69 |
| † calculated using the regression equations from Tables 10-12 in Sonmez et al. (2008). | | | |
